# Foodborne botulism from consumption of homemade spoiled eggs: a case series and literature review

**DOI:** 10.3389/fmed.2025.1649424

**Published:** 2025-09-29

**Authors:** Suyu Wei, Liang Sun

**Affiliations:** Department of Emergency, Shandong Provincial Third Hospital, Shandong University, Jinan, China

**Keywords:** botulism, poisoning, homemade food, antitoxin, case report

## Abstract

Botulism is a rare but life-threatening condition that primarily results from ingestion of food contaminated with the exotoxins produced by *Clostridium botulinum*. Although uncommon in clinical settings, it is characterized by acute onset, severe manifestations, and a high mortality rate. Outbreaks linked to egg consumption are rarely reported, and cases occurring during pregnancy are even less common, posing unique diagnostic and therapeutic challenges. We report a family cluster of botulism in China that was associated with the consumption of homemade fermented eggs. All affected individuals exhibited symptoms indicative of botulism, including vomiting, dysphagia, restricted eye movement, progressive limb muscle weakness, and other neurological deficits. Electromyography revealed evidence of neuromuscular dysfunction, and laboratory testing confirmed the presence of *C. botulinum* type A toxin, establishing the diagnosis of foodborne botulism. Both patients received timely supportive care, with one case requiring management during pregnancy. Antitoxin therapy was not administered due to delayed recognition, but both patients recovered fully and were discharged without complications. The pregnant woman subsequently delivered without adverse maternal or neonatal outcomes, and no recurrence was observed during follow-up. Accurate diagnosis of botulism can be challenging and careful epidemiological assessment combined with laboratory confirmation is essential to properly identify and define these cases. These cases underscore the importance of early recognition, timely diagnosis, and prompt treatment in improving patient outcomes, particularly in pregnancy-associated botulism.

## 1 Introduction

Botulism is a rare, neurotoxin-mediated, life-threatening condition caused by toxins produced by the anaerobic, Gram-positive bacterium *Clostridium botulinum* (1). The reported lethal dose of pure crystalline botulinum toxin type A for a 70-kg adult is estimated to be 70 μg orally and 0.80–0.90 μg by inhalation (2). Food contaminated with viable *C. botulinum* spores can become can become hazardous when stored under conditions that favor bacterial growth, such as low oxygen, low acidity (pH > 4.5), low salt and sugar content, and temperatures ranging from 37 to 99°F (3 to 37 °C) (3). Botulinum neurotoxins are produced by the several *Clostridium* species. Eight immunologically distinct serotypes have been identified (A, B, C, D, E, F, G, and X), with type A being the most virulent (4, 5). Clinical manifestations of poisoning are typically characterized by flaccid, descending paralysis that often begins with cranial nerve palsy and progresses to limb weakness and respiratory failure. The principal mechanism of toxicity is the inhibition of acetylcholine release at the neuromuscular junction (3). Outbreaks of foodborne botulism are typically associated with improperly processed homemade or fermented foods (6). Although most outbreaks affect only a small number of individuals, large-scale outbreaks can occur, making foodborne botulism a public health emergency (3). Diagnosis depends on a high index of clinical suspicion combined with thorough neurological examination. Timely diagnosis is critical, as botulinum antitoxin is the only specific treatment available.

Several foodborne botulism outbreaks have been reported worldwide. In 1978, an outbreak was reported among nomadic people in Kenya, where traditionally prepared milk contaminated with botulinum neurotoxin type A resulted in the death of six individuals (7). In February 2002, two siblings in South Africa, including a child, died of acute flaccid paralysis due to preformed toxin in commercially produced canned food (8). In 1999, 15 cases of foodborne botulism were reported in Morocco (9). Between 1959 and 2022, Qinghai Province in China reported 86 incidents, involving 348 cases and 195 deaths, corresponding to a case fatality rate of 56.03% (10). In 2021, two cases of botulism requiring treatment and diagnosis were reported in Hong Kong, China (11).

In our country, cases are most often associated with leavened beans or flour products. Contamination has also been detected in canned foods, dairy products, vacuum-packed products and frozen foods. However, outbreaks due to eggs appear to be rare and underreported. Moreover, reports of botulism in pregnancy are scarce, and evidence regarding maternal-fetal outcomes is limited, presenting unique diagnostic and therapeutic challenges. Here, we report a case of foodborne botulism following the collective consumption of homemade rotten eggs in our department. Based on our observations, the objective of this report is to describe the clinical course and diagnostic challenges, including in a pregnant patient, and to underscore the importance of early recognition and timely management in preventing complications.

## 2 Case presentation

### 2.1 Medical history and physical examination

**Figure 1:**
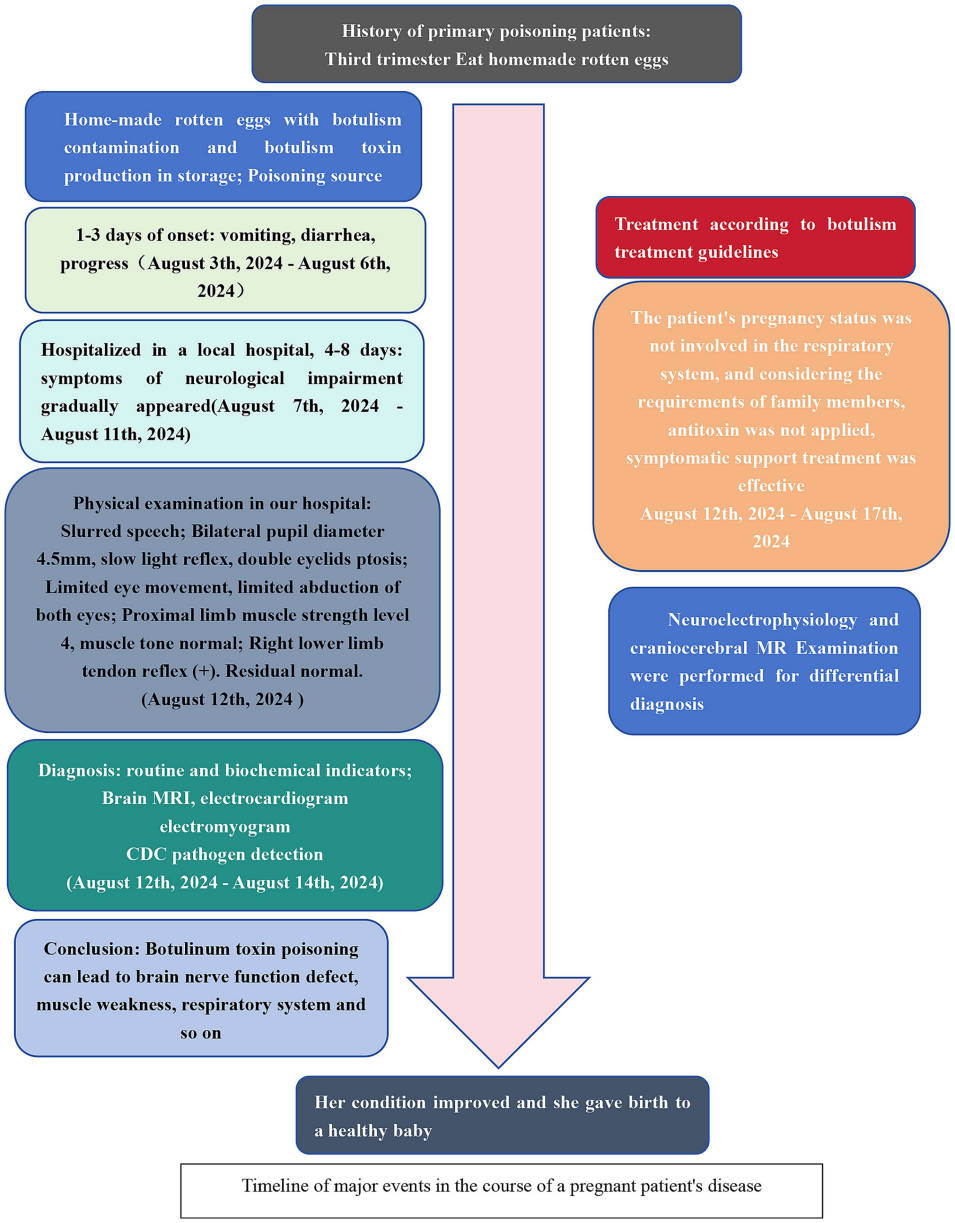
Flow chart.

**Figure 2:**
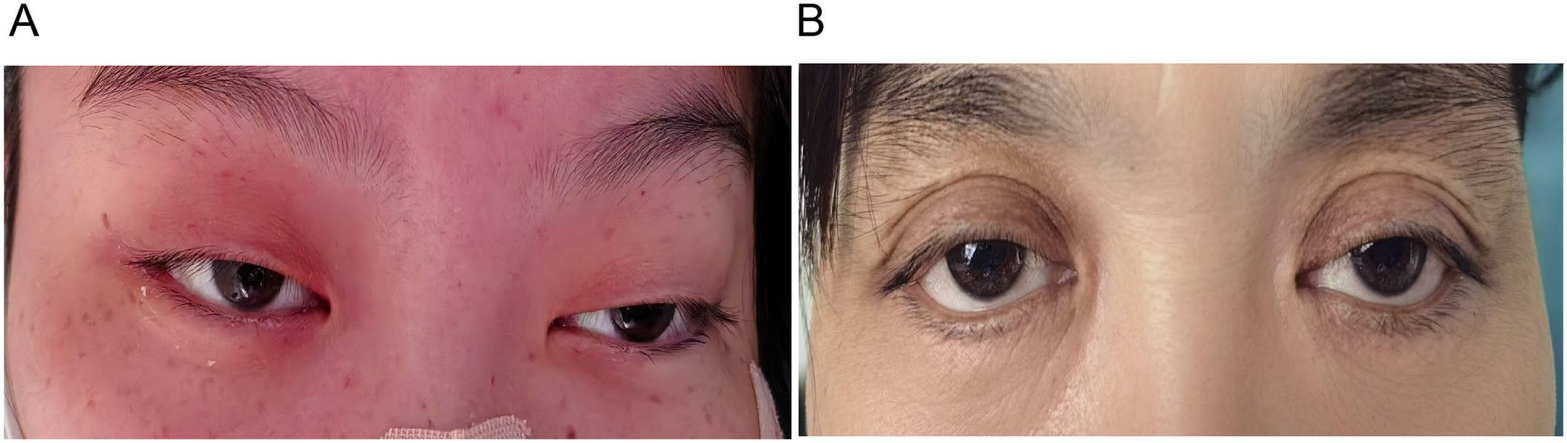
Eye status: **(A)** eye status of Patient A; **(B)** patient B’s eye status.

Patient A, a 23-year-old pregnant woman at >35 weeks gestation, was admitted to our hospital on August 12, 2024, with a chief complaint of “intractable vomiting for 3 days, ptosis and weakness of the extremities for 1 week” (Figure 1). Approximately 1 week prior to admission, she experienced postprandial vomiting after consuming homemade spoiled eggs. During the course of her illness, she developed a high-grade fever followed by bilateral ptosis and dysphagia. Three days before admission, her condition worsened, with new-onset limb weakness accompanied by dysphagia and dysarthria, restricted eye movements, and gait instability. She was initially treated at a local hospital, where she received comprehensive care including fluid replacement, electrolyte balance management, and protection of organs. However, the underlying cause was not identified, and treatment was ineffective. She was subsequently referred to our hospital for further evaluation. Based on a detailed review of her medical history, the likely cause of her condition was suspected to be foodborne botulism resulting from the ingestion of improperly preserved homemade eggs. On physical examination, her vital signs were as follows: body temperature, 37.0 °C; pulse rate, 73 beats per minute; respiratory rate, 19 breaths per minute; and blood pressure, 110/75 mmHg. Chest examination revealed symmetrical contours with clear breath sounds in both lungs, without dry or wet rales or wheezes. Cardiac examination showed no protrusion or depression in the precordial area, a normal apical impulse, regular heart rate, and no murmurs heard in any valvular areas. Bilateral arterial pulses were symmetrical, regular, and of moderate intensity. Abdominal examination showed distension without rash, hyperpigmentation, or varicose veins on the abdominal wall. There was no tenderness, rebound tenderness, or abdominal vascular murmurs, and bowel sounds were normal. Neurological examination revealed that pupils were isochoric, measuring approximately 5 mm in diameter, with sluggish response to light stimulation and bilateral ptosis graded at level IV-V (Figure 2A). Restricted ocular motility was observed bilaterally, with marked limitation of abduction. Deep tendon reflexes were normal in both upper limbs but exaggerated in the right lower limb; no responses were elicited in the left lower limb. Her family history was negative for hereditary disorders and infectious diseases. No abnormalities were noted in her psychological history.

Patient B, a 47-year-old woman, presented with complaints of dizziness, blurred vision, and dysphagia persisting for 10 days. Symptoms began following ingestion of homemade rotten eggs. Initially, she experienced vertigo and visual impairment, followed by nausea and vomiting. Her condition progressively worsened, leading to bilateral ptosis, dysphagia, limb weakness, and slurred speech. On preliminary examination, pupils were round, equal bilaterally at approximately 4 mm in diameter, and sluggishly responsive to light (Figure 2B). Her family history was negative for hereditary or infectious diseases.

### 2.2 Inspection and diagnosis basis

**Figure 3:**
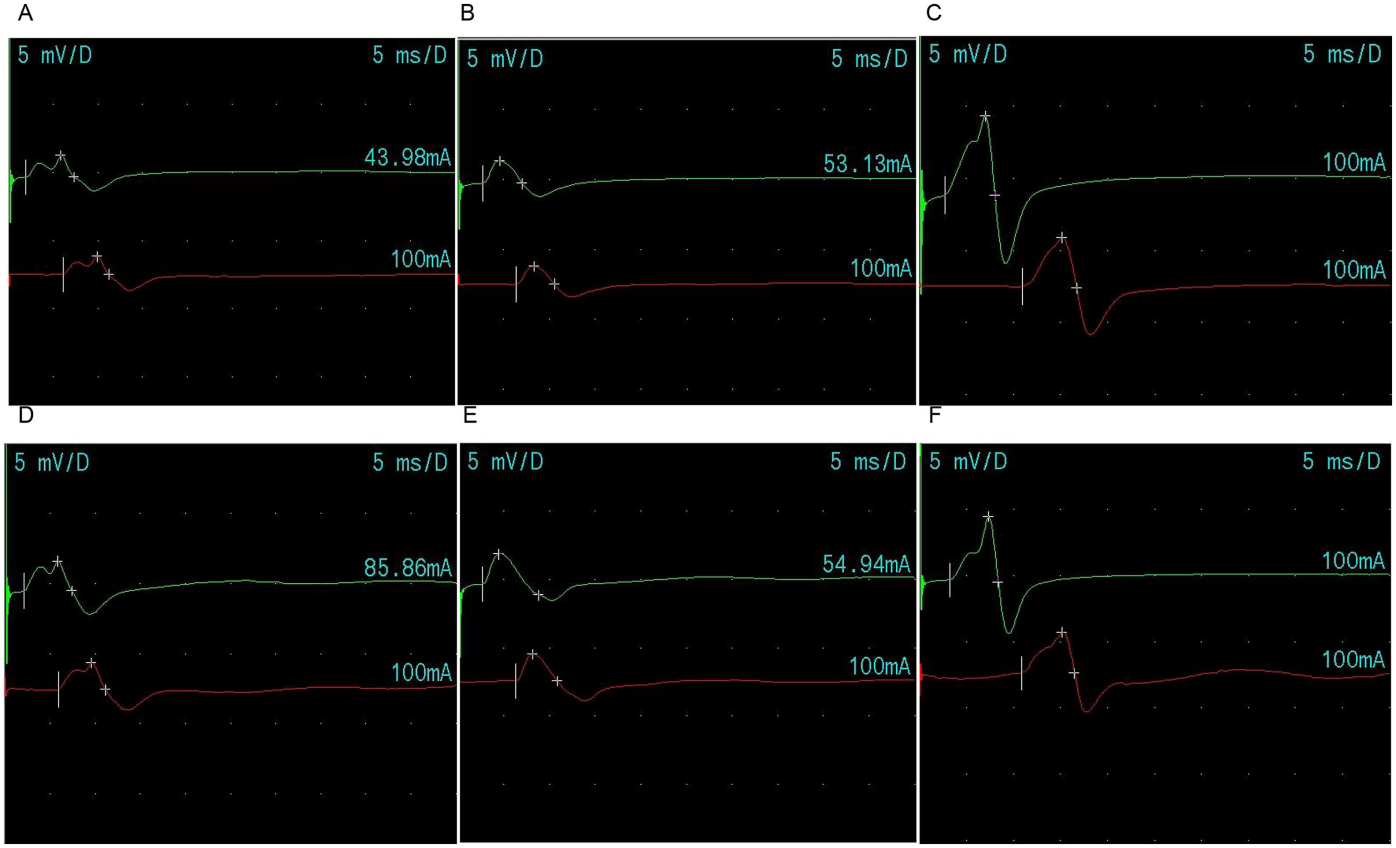
Electromyography results: **(A)** right ulnar nerve damage; **(B)** right median nerve damage; **(C)** right tibial nerve normal; **(D)** left ulnar nerve damage; **(E)** left median nerve damage; **(F)** left tibial nerve normal.

**Figure 4:**
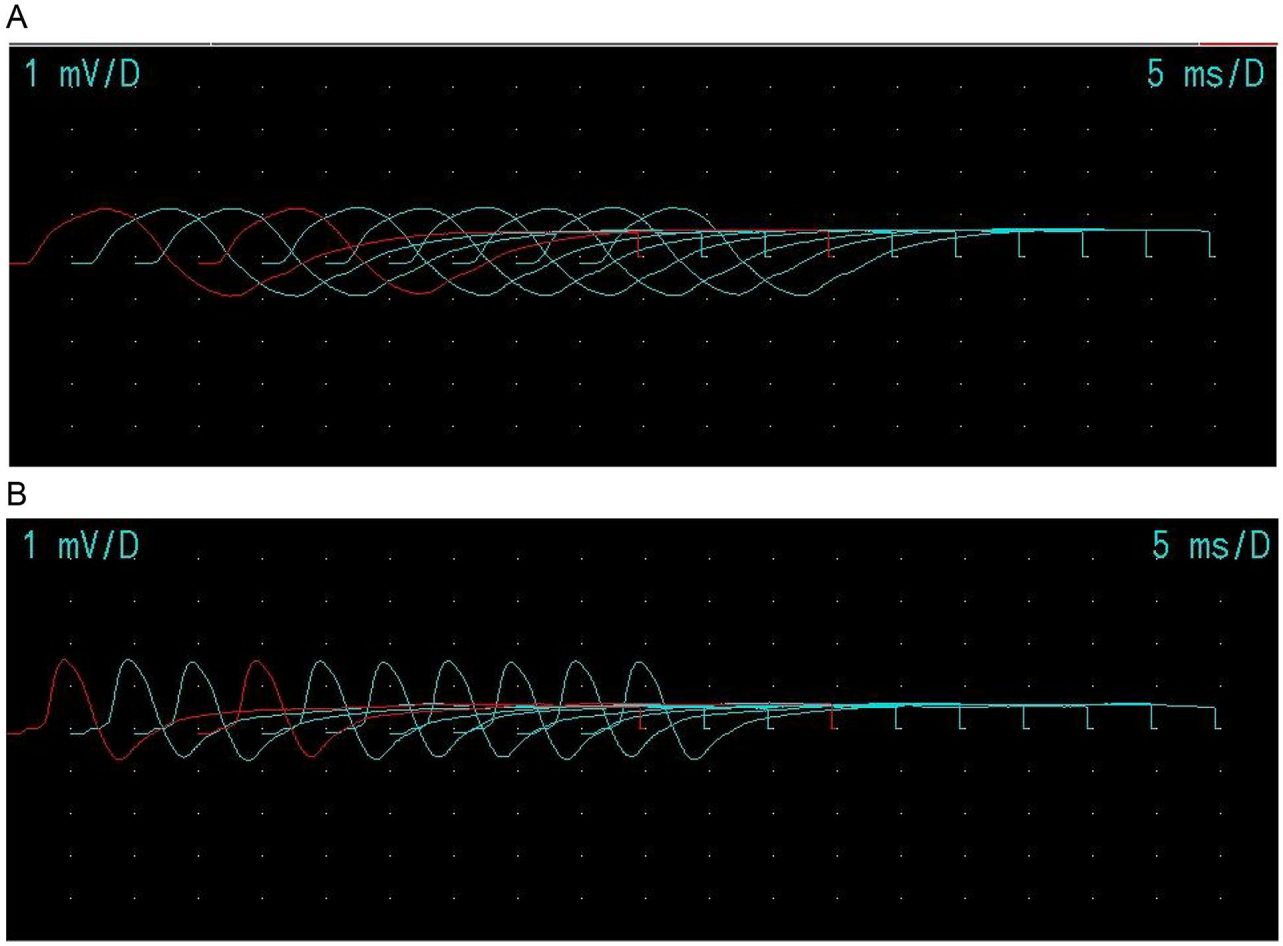
Electromyography results: **(A)** low-frequency stimulation; **(B)** high frequency stimulation.

Biochemical tests revealed hyponatremia, hypokalemia, and hyperchloremia. Electromyography (EMG) showed damage to the motor branches of the bilateral median and ulnar nerves, with abnormal F-waves (late motor responses) in both nerves (Figure 3). Low-frequency stimulation induced muscle contractions with atypical traits (Figure 4). The examination was interrupted due to limb tremors and uterine contractions in the late stage of pregnancy, and thus no images were captured during high-frequency stimulation. Food samples (“Homemade rotten eggs”) submitted for testing at the Center for Disease Control and Prevention yielded a positive result for *C. botulinum* type A. The patient developed symptoms 12 h after ingestion. During the initial phase, she remained conscious and exhibited no sensory abnormalities. Subsequently, she developed ptosis, restricted extraocular muscle movement, symmetrical pupil dilation, and delayed pupillary light reflexes. In the later phase, dysphagia, limb weakness, and slurred speech were noted. Cranial magnetic resonance imaging (MRI) showed no abnormalities. Based on the characteristic clinical presentation and detection of botulinum neurotoxin type A, the case was definitively diagnosed as foodborne botulism.

### 2.3 Differential diagnoses

Differential diagnoses considered for this patient included: (i) Guillain–Barré Syndrome (GBS) which typically presents with ascending paralysis (lower limb → trunk → upper limbs → respiratory muscles), often accompanied by sensory abnormalities such as limb numbness or tingling. Cerebrospinal fluid analysis usually shows protein-cell dissociation. However, this patient exhibited only ocular muscle paralysis without sensory abnormalities like limb numbness, and EMG findings were inconsistent with GBS. (ii) Myasthenia Gravis which is characterized by worsening symptoms in the evening and improvement in the morning. It is often triggered by infection, fatigue, or medications (e.g., aminoglycoside antibiotics). Symptoms typically improve after neostigmine (a cholinesterase inhibitor) injection. In this case, the patient exhibited no morning improvement, had a significant history of consuming contaminated food, and later developed symptoms consistent with botulism, including dysphagia. Additionally, all five diagnostic tests for myasthenia gravis were negative. (iii) Cerebral infarction or hemorrhage which usually represents with altered consciousness (somnolence or coma) and pupillary abnormalities (e.g., pinpoint pupils, anisocoria), with characteristic findings on cranial computed tomography (CT) or MRI. In this patient, cranial MRI was normal, effectively ruling out cerebral infarction or hemorrhage.

### 2.4 Treatment measures and outcomes

Upon admission, both patients received immediate oxygen therapy, nasogastric feeding (enteral nutrition suspension, 500 mL once daily for 5 days; fat emulsion injection, 100 mL once daily for 5 days), anti-infective therapy (cefazolin, 4 g once daily for 4 days), and electrolyte replacement (5% glucose injection, 500 mL plus potassium chloride injection, 2 g once daily for 5 days; 0.9% sodium chloride injection, 500 mL plus concentrated sodium chloride injection, 2 g once daily for 5 days). In addition, low-dose glucocorticoids (dexamethasone sodium phosphate injection, 5 mg twice daily for 1 day), high-dose vitamins (0.9% sodium chloride injection, 500 mL, vitamin C injection, 2 g, vitamin B6 injection, 0.2 g, and potassium chloride, 1 g once daily for 7 days), and drugs to protect vital organs such as the heart, brain, liver, kidney, and gastric mucosa were administered. The pregnant patient received obstetric consultation and was advised to undergo symptomatic treatment with botulinum antitoxin type A injection. The other patient was also recommended for antitoxin therapy. However, after careful consideration, both families refused antitoxin administration. The pregnant woman and her relatives believed that the injection might pose a risk to fetal health and development. They also felt that because the patient was gradually recovering, antitoxin administration was unnecessary. Therefore, they insisted on conservative management and declined antitoxin therapy. Neither patient experienced significant respiratory compromise during treatment, thus requiring no non-invasive ventilation or endotracheal intubation with mechanical ventilation support. Both maintained oxygen saturation levels between 96 and 99% with stable blood pressure. Pulmonary examination revealed no dry or wet rales, wheezes, or rhonchi. Extraocular muscle movements returned to normal, and both patients exhibited no dysphagia or choking while consuming liquids. Limb mobility showed no significant impairment, with muscle strength graded as 5. Pupils were equal in size, light-reactive, with diameters of 2.5 mm and intact pupillary light reflexes. Patients were able to ambulate independently and reported no nausea, vomiting, or abdominal pain. Throughout the hospitalization, no antitoxin was administered, and therefore, no adverse events related to antitoxin allergy occurred. After symptomatic treatment, both patients improved and were discharged without any sequelae. The pregnant patient delivered without complications, and the neonate healthy. The patient and their family members expressed their gratitude to the medical staff when they were discharged from the hospital.

### 2.5 Post-discharge follow-up

Three months after discharge, we conducted a telephone follow-up to assess the health status of the patient and the infant. The patient and her family reported no symptoms. The patient was able to move independently and the mobility of her limbs remained unimpaired. We advised the patient and the infant to visit the neurology department and other relevant hospital departments for a comprehensive examination.

### 2.6 Public health measures

Home-prepared foods are a major cause of botulism, often resulting in sudden public health emergencies. Therefore, enhancing food safety education and preventive measures to home-prepared foods is essential. Through targeted food safety educational programs, the public can better understand risk factors and identify high-risk foods. Simultaneously, implementing preventive measures—strict control of ingredients, thorough processing, and standardized storage—can significantly reduce the risk of poisoning. This approach ensures home-prepared foods retain their flavor while maintaining safety and health benefits.

## 3 Discussion

Botulism is a rare disease of the neuromuscular junction caused by the anaerobic, Gram-positive rod-shaped bacterium *C. botulinum* (1, 12). Endospores of *C. botulinum* are widely distributed in soils worldwide, and their spores can survive indefinitely, including resistance to heating that inactivates vegetative bacterial cells (13). Botulism may result from ingestion of contaminated fermented or spoiled food, production of botulinum toxin by toxic bacilli in the intestine or wounds, or inhalation of aerosolized botulinum toxin (14). The early characteristic symptoms of foodborne botulism include acute onset of neurological deficits such as inattention and ptosis, followed by bilateral, symmetrical, descending flaccid paralysis, without sensory disturbance. Paralysis typically begins with the cranial nerves and progresses to involve the respiratory muscles, upper and lower limbs, potentially leading to respiratory failure and death (15–17). The pathophysiology of botulism is mediated by botulinum neurotoxin, which blocks presynaptic acetylcholine release at the neuromuscular junction. This inhibition prevents muscle contraction, leading to flaccid paralysis (18, 19).

With the advancement of medical technology, botulinum toxin (commonly known as Botox) is widely used in cosmetic industry, and the incidence of foodborne botulism is gradually decreasing. Therefore, when considering botulism, it is important to rule out other neuromuscular disorders, including GBS, myasthenia gravis, Lambert–Eaton myasthenic syndrome, stroke, and opioid overdose (20, 21). Botulinum toxin is an exotoxin that is extremely toxic, with fatal outcomes possible in minute amounts. Early administration of antitoxin, ideally within 24 h of symptom onset, reduces morbidity and mortality.

Both patients in this report were initially hospitalized at a local medical facility but were not correctly diagnose with botulism in a timely manner. This led to a delay in antitoxin treatment and a missed opportunity for optimal intervention. This case underscores the importance of prompt recognition of botulism in clinical practice. Diagnosis was based on a history of suspicious food consumption, typical clinical manifestations, and laboratory findings. Detection of botulinum toxin in patient’s serum remains the most direct and reliable diagnostic method. Additionally, the detection of toxin in stool samples aids in clinical confirmation, while testing food samples is essential for establishing the link between ingestion and poisoning, as well as verifying the accuracy of clinical diagnosis. These observations highlight the critical role of detailed medical history collection in clinical practice.

The present case involved a family cluster in which affected individuals developed nausea, vomiting, and abdominal pain after consuming homemade fermented eggs. These symptoms are consistent with the gradual onset of foodborne botulism and the gastrointestinal toxicity it induces. EMG findings indicated that the patient suffered from neuromuscular damage, a condition that aligns with the clinical symptoms of dysphagia and limb weakness due to impaired muscle contraction. These findings align with previously reported cases. In this instance, two patients consumed homemade rotten eggs simultaneously and became ill, which strongly supported the clinical suspicion of botulism and was consistent with the existing literature. Although rare, foodborne botulism should be considered in patients presenting with cranial nerves dysfunction and quadriplegia of unclear etiology. Importantly, one of the affected patients was pregnant, requiring careful consideration in both diagnosis and management. During testing, contractions were induced by low-frequency stimulation, and the patient experienced limb tremors with high-frequency stimulation that interrupted the examination. These findings illustrate the limitations of diagnostic testing in pregnant patients. Therefore, clinicians should integrate patient’s medical history, the course of the disease, and the relevant auxiliary tests promptly to ensure accurate diagnosis and timely treatment in pregnancy-associated cases of botulism. Furthermore, in this case, *C. botulinum* did not accumulate in the respiratory system, and the patient recovered with symptomatic supportive treatment. Notably, the mother subsequently had an uneventful delivery. Clinical observation and assessment revealed that the infant did not exhibit any specific symptoms, signs, or abnormal organ functions related to botulism, suggesting that botulism did not have a significant impact on the infant. The botulinum toxin did not cross the placental barrier, ensuring the neonate remained unaffected. In a case involving a family meal, the pregnant woman experienced early onset of symptoms, which appeared approximately 8 days after her mother’s arrival. She recovered swiftly following symptomatic treatment. Following targeted treatment, both patients showed improvement and were discharged without complications. This favorable outcome may be attributed to several factors: (i) the low dose of toxin ingested, which affected only superficial muscle groups without involving respiratory muscles; (ii) the patients’ young age and absence of underlying medical conditions, which facilitated strong neural regeneration and compensatory physiological capacity; and (iii) the comprehensive supportive care during treatment, which prevented secondary complications. However, current guidelines do not clearly indicate whether shared meals, the timing of symptom onset, or the severity of symptoms are linked to pregnancy. Further research and observation are required to establish these connections. This case highlights the potential increased susceptibility of pregnant women to infection and more severe disease, underscoring the importance of early detection and prompt management in such cases. Both physicians and patients must be aware of the importance of preventing and managing botulism. For patients, health education should be strengthened to improve awareness of botulism prevention. For clinicians, timely, accurate, and appropriate interventions are essential. Once early-stage auxiliary examinations are completed, blood, stool and suspicious food sample should be collected promptly for botulinum toxin detection and analysis. Where feasible, aerobic and anaerobic cultures and isolation of bacteria from stool and food sample, should be performed to confirm the etiology. Following confirmation of botulinum toxin poisoning, prompt administration of a high dose of anti-botulinum toxin is recommended. In cases where the toxin type is not classified, simultaneous injection of type A and B anti-botulinum toxins may improve treatment outcomes. This study has certain limitations. First, delayed diagnosis occurred because botulinum toxin poisoning was not promptly identified during initial treatment at local hospitals after symptom onset. Second, patient biological samples were not collected for monitoring in a timely manner, and family members may have introduced recall bias when reporting clinical symptoms. Third, one patient was pregnant, which restricted the use of botulinum antitoxin and other medications.

## 4 Conclusion

In conclusion, for patients with suspected botulism, clinicians should early identification, diagnosis, and treatment, which for the prognosis of patients.

## Data Availability

The raw data supporting the conclusions of this article will be made available by the authors, without undue reservation.
